# Multidrug-resistant Gram-negative clinical isolates with reduced susceptibility/resistance to cefiderocol: which are the best present and future therapeutic alternatives?

**DOI:** 10.1007/s10096-023-04732-4

**Published:** 2023-12-14

**Authors:** Christophe Le Terrier, Samanta Freire, Patrice Nordmann, Laurent Poirel

**Affiliations:** 1 Emerging Antibiotic Resistance Unit, Medical and Molecular Microbiology, Department of Medicine, University of Fribourg, Chemin du Musée 18, CH-1700 Fribourg, Switzerland; 2 Division of Intensive Care Unit, University hospitals of Geneva, Geneva, Switzerland; 3 Swiss National Reference Center for Emerging Antibiotic Resistance, Fribourg, Switzerland

**Keywords:** Cefiderocol, Taniborbactam, Avibactam, Nacubactam, Zidebactam, Relebactam, Vaborbactam, β-Lactamase

## Abstract

**Purpose:**

To evaluate the different present and future therapeutic β-lactam/β-lactamase inhibitor (BL/BLI) alternatives, namely aztreonam-avibactam, imipenem-relebactam, meropenem-vaborbactam, cefepime-zidebactam, cefepime-taniborbactam, meropenem-nacubactam, and sulbactam-durlobactam against clinical isolates showing reduced susceptibility or resistance to cefiderocol in Enterobacterales, *Acinetobacter baumannii*, and *Pseudomonas aeruginosa.*

**Methods:**

MIC values of aztreonam, aztreonam-avibactam, cefepime, cefepime-taniborbactam, cefepime-zidebactam, imipenem, imipenem-relebactam, meropenem, meropenem-vaborbactam, meropenem-nacubactam, sulbactam-durlobactam, and cefiderocol combined with a BLI were determined for 67, 9, and 11 clinical Enterobacterales, *P. aeruginosa* or *A. baumannii* isolates, respectively, showing MIC values of cefiderocol being ≥1 mg/L. If unavailable, the respective β-lactam breakpoints according to EUCAST were used for BL/BLI combinations.

**Results:**

For Enterobacterales, the susceptibility rates for aztreonam, cefepime, imipenem, and meropenem were 7.5%, 0%, 10.4%, and 10.4%, respectively, while they were much higher for cefepime-zidebactam (91%), cefiderocol-zidebactam (91%), meropenem-nacubactam (71.6%), cefiderocol-nacubactam (74.6%), and cefiderocol-taniborbactam (76.1%), as expected. For *P. aeruginosa* isolates, the higher susceptibility rates were observed for imipenem-relebactam, cefiderocol-zidebactam, and meropenem-vaborbactam (56% for all combinations). For *A. baumannii* isolates, lower susceptibility rates were observed with commercially or under development BL/BLI combos; however, a high susceptibility rate (70%) was found for sulbactam-durlobactam and when cefiderocol was associated to some BLIs.

**Conclusions:**

Zidebactam- and nacubactam-containing combinations showed a significant in vitro activity against multidrug-resistant Enterobacterales clinical isolates with reduced susceptibility to cefiderocol. On the other hand, imipenem-relebactam and meropenem-vaborbactam showed the highest susceptibility rates against *P. aeruginosa* isolates. Finally, sulbactam-durlobactam and cefiderocol combined with a BLI were the only effective options against *A. baumannii* tested isolates.

**Supplementary Information:**

The online version contains supplementary material available at 10.1007/s10096-023-04732-4.

## Introduction

The global spread of Gram-negative bacteria with pan-drug resistance is a worrying concern [1]. In 2017, the World Health Organization (WHO) ranked carbapenem-resistant Enterobacteriaceae (CRE), carbapenem-resistant *Pseudomonas aeruginosa*, and carbapenem-resistant *Acinetobacter baumannii* in the critical global priority list of pathogens [2]. This matter of concern has promoted research and development in the area of antibiotic resistance including the development of novel antibiotics. Acquired resistance to carbapenems may be driven either by non-enzymatic mechanisms including loss of or weaker expression of porin-encoding genes, mutations in chromosomally encoded porin genes (such as OprD in *P. aeruginosa*, or OmpK35 and OmpK36 in *Klebsiella pneumoniae*), overexpression of genes encoding efflux pumps, or modifications of penicillin-binding proteins affecting the binding affinity of the β-lactams, particularly in *A. baumannii* [3–6]. However, the main concern regarding the mechanism of carbapenem resistance is the production of acquired carbapenemases, belonging to either Ambler class A (mainly KPC and GES), class B (i.e., NDM, VIM, IMP), or class D (i.e., OXA-23, OXA-24/40, OXA-48, and OXA-181) β-lactamases, particularly found in Enterobacterales or *A. baumannii.* Considering that those different resistance mechanisms are often combined, very few therapeutical options may sometimes be left, especially when dealing with infections caused by producers of metallo-β-lactamases (class B).

The new siderophore cephalosporin cefiderocol is one of the few antibiotics being considered for treating infections caused by these so-called difficult-to-treat (DTR) Gram-negative pathogens, since this molecule is not significatively hydrolyzed by most β-lactamases, including carbapenemases. In addition, cefiderocol bactericidal activity can overcome most non-enzymatic resistance mechanisms (porin mutations, and efflux pumps) [7]. Nevertheless, several mechanisms of resistance to cefiderocol have been recently reported, such as porin mutations, mutations affecting siderophore receptors, efflux pump overproduction, and modifications of the target (penicillin binding protein 3, or PBP-3), or production of some given β-lactamases [8]. A wide range of β-lactamases including class A (PER, BEL, SHV, KPC), class B (NDM, VIM), class C (AmpC), or class D β-lactamases (OXA-2, OXA-10, OXA-46) has been demonstrated to contribute to reduce the susceptibility to cefiderocol [8–11]. In addition, the progressive in vivo development of reduced susceptibility to cefiderocol has been reported in clinical contexts, particularly within two clinical trials [12, 13].

Recently, many promising β-lactamase inhibitors have been developed and might be soon clinically available along with respective β-lactam partner molecules, providing novel clinically relevant β-lactamase/β-lactamase inhibitor (BL/BLI) combos, useful to treat infections caused by extensively drug-resistant strains [14]. Among the newly developed BLIs, there are diazabicyclooctane (DBO) molecules, namely zidebactam, durlobactam, and nacubactam, that efficiently inhibit most class A and C (also some class D for zidebactam and durlobactam) β-lactamases, and additionally, they exhibit effective antibacterial activity by targeting PBP-2, in contrast to the presently available DBO avibactam, which have a weaker antibacterial activity. Another class of inhibitors corresponds to the boronic acid derivatives, with taniborbactam inhibiting the hydrolytic activity of class A and C β-lactamases, but also that of metallo-beta-lactamases (MBLs). Taniborbactam inhibits most NDM- and VIM-type β-lactamases (except NDM-9, NDM-30, and VIM-83), but does not show significant inhibitory activity against IMP-type enzymes [15–18]. Altogether, the development of all those new BLIs has promoted the development and the evaluation of novel BL/BLI combinations including aztreonam-avibactam, cefepime-zidebactam (WCK 5107, DBO), cefepime-taniborbactam (VNRX-5133, boronate), meropenem-nacubactam (FPI-1465, DBO), and sulbactam-durlobactam (ETX-2514) which have been tested in several in vitro studies and are now undergoing clinical evaluations, while imipenem-relebactam and meropenem-vaborbactam are already clinically-available [19–27].

The objective of our study was therefore to evaluate the in vitro activity of these new combos against multidrug-resistant Gram-negative isolates showing reduced susceptibility to cefiderocol, and to evaluate the potential benefit of adding a BLI (avibactam, relebactam, nacubactam, zidebactam, or taniborbactam) together with cefiderocol.

## Material and methods

### Bacterial isolates

A collection of 67 multidrug-resistant Enterobacterales, nine *P. aeruginosa*, and eleven *A. baumannii* clinical isolates showing reduced susceptibility to cefiderocol (MIC value ≥1 mg/L) determined by broth microdilution method using iron depleted Mueller-Hinton that had been collected by the Swiss National Reference Center for Emerging Antibiotic Resistance (NARA) across all Switzerland was used for this study [28]. Clinical isolates included *Escherichia coli* (*n*= 28), *Klebsiella pneumoniae* (*n*=19), *Enterobacter cloacae* (*n*=12), *Citrobacter freundii* (*n*=6), *Klebsiella oxytoca* (*n*=2), *P. aeruginosa* (*n*=9), and *A. baumannii* (*n*=11). Most of those isolates produced an MBL, including NDM-like (*n*=61), or VIM-like (*n*=6) and had previously been characterized at the molecular level (Table 1). Most of these isolates additionally produced extended-spectrum β-lactamases (Table 1). Our collection included also 25 non-duplicate *E. coli* strains possessing a four amino-acid insertion (YRIN or YRIK) in their PBP3 protein sequence that had been previously found to be less susceptible or resistant to the aztreonam-avibactam combination [22].

**Table 1:** MIC distribution of novel BL/BLI under clinical development based on aztreonam, cefepime, imipenem, and meropenem, associated to cefiderocol in the presence and absence of avibactam, relebactam, zidebactam, taniborbactam, and nacubactam for 67 cefiderocol-resistant *Enterobacterales* strains, 9 cefiderocol-resistant *Pseudomonas aeruginosa* strains, and 11 cefiderocol-resistant *Acinetobacter baumannii* strains

Clinical isolates species	ST	Acquired β-lactamases content^b^	PBP3^c^ modified	Minimal inhibitory concentration (mg/L)^a^																			
				FDC	ATM	AZA	FEP	FEP-TAN	FEP-ZID	IPM	I/R	MEM	MVB	MEM-NAC	FDC-AVI	FDC-REL	FDC-TAN	FDC-ZID	FDC-VAB	FDC-NAC	ZID	NAC	SUL-DUR
*E. coli*	167	**NDM-19**	YRIN	16	0.5	0.5	256	16	≤0.125	8	8	32	16	≤0.125	32	32	4	≤0.125	8	32	0.25	1	–
*E. coli*	ND	**NDM-1**	–	4	>256	2	128	16	≤0.125	8	8	32	32	≤0.125	≤0.125	≤0.125	0.25	≤0.125	2	≤0.125	0.5	2	–
*E. coli*	ND	**NDM-1**, CTX-M-15	–	1	128	0.25	>256	2	≤0.125	4	4	4	2	2	≤0.125	≤0.125	≤0.125	≤0.125	1	≤0.125	0.125	>16	–
*E. coli*	ND	**NDM-1**, CTX-M-15	–	32	32	≤0.125	>256	2	≤0.125	8	8	16	8	4	4	16	0.5	≤0.125	8	0.25	0.03	1	–
*E. coli*	ND	**NDM-5,** CTX-M-15	YRIN	32	>256	1	>256	32	≤0.125	16	16	64	16	≤0.125	32	16	1	0.5	8	16	0.125	1	–
*E. coli*	167	**NDM-7,** CTX-M-15	YRIN	128	>256	0.5	>256	64	≤0.125	16	32	64	32	≤0.125	128	128	32	≤0.125	128	≤0.125	0.125	1	–
*E. coli*	ND	**NDM-6,** CTX-M-15	YRIN	32	>256	1	>256	32	≤0.125	8	8	32	16	≤0.125	16	32	8	≤0.125	32	≤0.125	0.125	1	–
*E. coli*	ND	**NDM-1,** CTX-M-15	YRIN	32	>256	1	>256	32	≤0.125	8	8	64	64	≤0.125	16	32	8	0.25	32	1	0.125	1	–
*E. coli*	ND	**NDM-5,** CTX-M-15	YRIN	64	>256	1	>256	128	≤0.125	16	16	128	32	≤0.125	16	8	16	0.5	32	1	0.5	1	–
*E. coli*	ND	**NDM-6,** CTX-M-15	YRIN	64	>256	1	>256	32	≤0.125	4	4	32	32	≤0.125	64	64	16	32	16	8	0.125	1	–
*E. coli*	ND	**NDM-7,** CTX-M-15	YRIN	128	>256	1	>256	64	≤0.125	32	32	64	32	64	128	128	32	0.25	128	0.25	0.125	1	–
*E. coli*	ND	**NDM-7**	YRIN	16	>256	≤0.125	>256	32	≤0.125	32	32	64	64	≤0.125	8	16	2	≤0.125	16	0.25	0.25	1	–
*E. coli*	167	**NDM-1**	YRIN	>256	64	16	>256	64	≤0.125	16	16	64	32	≤0.125	>256	>256	>256	>256	128	>256	0.25	2	–
*E. coli*	ND	**NDM-1**	YRIN	8	2	1	>256	32	≤0.125	64	64	128	64	≤0.125	4	8	2	≤0.125	8	2	0.125	1	–
*E. coli*	410	**NDM-5,** CTX-M-15	YRIK	2	>256	4	>256	16	≤0.125	8	8	8	8	4	0.25	0.5	≤0.125	≤0.125	4	≤0.125	0.125	1	–
*E. coli*	405	**NDM-5,** CTX-M-15, OXA-1	YRIK	2	>256	8	>256	64	≤0.125	8	8	16	16	≤0.125	1	2	0.25	≤0.125	2	0.25	0.125	2	–
*E. coli*	617	**NDM-5,** CTX-M-15	YRIK	8	>256	2	8	64	≤0.125	8	8	16	8	≤0.125	0.25	1	0.25	≤0.125	8	≤0.125	0.125	1	–
*E. coli*	ND	**NDM-5,** CTX-M-15	YRIN	32	>256	2	>256	64	≤0.125	32	32	64	64	128	8	16	4	≤0.125	32	0.25	0.5	16	–
*E. coli*	ND	**NDM-5**	YRIN	32	64	16	>256	64	≤0.125	16	16	64	32	128	16	32	8	4	128	16	0.25	4	–
*E. coli*	ND	**NDM-5,** OXA-181	YRIN	16	0.5	0.5	128	8	≤0.125	8	8	16	16	≤0.125	4	8	2	≤0.125	16	≤0.125	0.06	1	–
*E. coli*	ND	**NDM-5**	YRIN	2	16	2	>256	32	≤0.125	8	8	32	16	≤0.125	0.5	0.5	0.5	≤0.125	2	1	0.125	2	–
*E. coli*	ND	CTX-M-15	YRIK	2	>256	16	>256	16	≤0.125	0.5	0.25	1	≤0.125	0.5	≤0.125	0.25	0.25	≤0.125	1	≤0.125	1	1	–
*E. coli*	ND	**NDM-5,** CTX-M-15	YRIK	4	>256	4	>256	64	≤0.125	8	8	16	16	≤0.125	1	4	1	≤0.125	8	≤0.125	0.06	1	–
*E. coli*	ND	**NDM-5**	YRIN	2	64	8	>256	64	≤0.125	16	16	64	32	64	2	2	0.5	≤0.125	2	2	0.5	4	–
*E. coli*	ND	**NDM-5**	YRIN	2	128	8	256	64	≤0.125	16	16	32	32	32	4	4	1	≤0.125	2	4	0.5	4	–
*E. coli*	ND	**NDM-5,** CTX-M-15	YRIN	16	>256	2	>256	64	≤0.125	16	16	64	16	64	8	16	1	≤0.125	16	16	0.5	16	–
*E. coli*	ND	**NDM-5,** CTX-M-15	YRIN	16	>256	≤0.125	>256	>128	≤0.125	32	32	128	32	≤0.125	8	16	1	≤0.125	16	4	0.25	1	–
*E. coli*	ND	**NDM-5,** CTX-M-15	YRIN	2	64	8	>256	64	≤0.125	16	16	64	32	64	2	2	0.5	≤0.125	2	2	0.5	4	–
*K. pneumoniae*	ND	KPC-2**,** SHV-11	ND	8	>256	≤0.125	>256	64	≤0.125	256	≤0.125	>256	64	≤0.125	1	2	2	0.5	4	1	4	2	–
*K. pneumoniae*	2096	**NDM-2,** CTX-M-15, SHV-28, OXA-9, TEM-1, OXA-1	A375V G192V	8	>256	0.5	>256	32	8	32	64	64	32	64	0.25	1	1	≤0.125	8	≤0.125	>16	>16	–
*K. pneumoniae*	15	**NDM-1**, SHV-28, CMY-6, CTX-M-15, TEM-1, OXA-1	ND	1	>256	>128	>256	>128	≤0.125	64	64	128	64	≤0.125	≤0.125	≤0.125	≤0.125	≤0.125	1	≤0.125	4	1	–
*K. pneumoniae*	14	**VIM-1**, SHV-5	A375V	32	>256	8	>256	128	>128	32	64	128	64	128	1	8	1	1	32	8	>16	>16	–
*K. pneumoniae*	147	**NDM-1**, SHV-11, TEM-32, OXA-1, OXA-9, CTX-M-15	A375V	1	>256	32	>256	16	128	64	64	128	64	128	≤0.125	0.5	≤0.125	≤0.125	1	≤0.125	>16	16	–
*K. pneumoniae*	512	**NDM-1,** KPC-3, SHV-11	ND	2	>256	≤0.125	>256	16	≤0.125	>256	32	>256	64	≤0.125	0.25	0.25	0.25	≤0.125	2	≤0.125	2	2	–
*K. pneumoniae*	11	**NDM-1**, OXA-181	ND	1	>256	0.25	>256	32	≤0.125	64	64	128	64	0.25	≤0.125	≤0.125	≤0.125	≤0.125	1	≤0.125	1	>8	–
*K. pneumoniae*	15	**NDM-1**, OXA-181, SHV-11, CTX-M-15, OXA-1	A375V	1	>256	128	>256	>128	>128	256	>128	256	64	>128	≤0.125	≤0.125	≤0.125	≤0.125	1	0.5	>16	>16	–
*K. pneumoniae*	ND	**NDM-1**, SHV-11, CTX-M-15	ND	1	>256	1	>256	128	≤0.125	64	64	128	64	≤0.125	≤0.125	≤0.125	≤0.125	≤0.125	1	≤0.125	2	1	–
*K. pneumoniae*	15	**NDM-1**, SHV-28, CMY-75, CTX-M-15	ND	2	64	≤0.125	64	1	≤0.125	4	4	4	4	4	0.25	≤0.125	≤0.125	≤0.125	2	≤0.125	0.06	1	–
*K. pneumoniae*	15	**NDM-1**, SHV-28, CMY-75, CTX-M-15	ND	2	32	≤0.125	>256	1	≤0.125	8	4	8	4	≤0.125	≤0.125	0.25	0.25	≤0.125	2	≤0.125	0.125	1	–
*K. pneumoniae*	ND	**NDM-1**, OXA-48, CTX-M-15	ND	2	>256	>128	>256	>128	>128	>256	>128	>256	64	>128	≤0.125	0.25	≤0.125	≤0.125	2	≤0.125	>16	>16	–
*K. pneumoniae*	101	**NDM-1**, OXA-48, CTX-M-15, OXA-9, TEM-1, SHV-100, SHV-45, SHV-198	ND	4	>256	≤0.125	>256	128	≤0.125	64	64	256	64	≤0.125	≤0.125	0.25	≤0.125	≤0.125	4	0.25	1	2	–
*K. pneumoniae*	147	**NDM-1**, SHV-11, TEM-1, OXA-9, CTX-M-224, CTX-M-54	ND	1	>256	≤0.125	>256	1	0.25	8	8	8	4	≤0.125	≤0.125	≤0.125	≤0.125	≤0.125	1	≤0.125	0.5	2	–
*K. pneumoniae*	268	**NDM-1**, SHV-11, CMY-4, TEM-1, OXA-10, CTX-M-15	A375V	2	128	≤0.125	>256	0.5	≤0.125	2	4	2	1	0.5	≤0.125	0.25	0.25	≤0.125	2	≤0.125	>16	>16	–
*K. pneumoniae*	ND	**NDM-1**, CTX-M-15	ND	2	128	≤0.125	>256	0.25	≤0.125	1	2	2	2	1	≤0.125	0.25	0.25	≤0.125	2	0.5	>16	8	–
*K. pneumoniae*	ND	**NDM-7**	ND	2	≤0,25	≤0.125	32	1	0.25	16	16	32	32	≤0.125	1	2	0.25	≤0.125	2	≤0.125	2	16	–
*K. pneumoniae*	ND	**NDM-1**, CTX-M-15	ND	4	64	≤0.125	>256	4	≤0.125	4	4	8	8	4	≤0.125	≤0.125	≤0.125	≤0.125	4	≤0.125	2	16	–
*K. pneumoniae*	ND	**NDM-1,** SHV-187	ND	4	64	≤0.125	>256	0.25	≤0.125	1	2	1	1	1	1	1	0.5	≤0.125	4	0.5	0.25	2	–
*K. oxytoca*	ND	**NDM-1,** SHV-28	ND	8	≤0.25	≤0.125	16	0.5	0.25	4	4	2	2	2	1	8	1	≤0.125	8	≤0.125	0.125	4	–
*K. oxytoca*	ND	**NDM-7**	ND	1	>256	≤0.125	>256	64	≤0.125	128	128	128	64	≤0.125	0.5	1	0.25	≤0.125	1	0.5	1	2	–
*C. freundii*	ND	**NDM-1**	ND	1	128	>128	32	4	≤0.125	4	4	4	4	≤0.125	1	≤0.125	0.25	1	1	≤0.125	1	1	–
*C. freundii*	ND	**NDM-1,** CMY-48, CTX-M-15, TEM-1, OXA-9	ND	1	64	≤0.125	>256	0.5	1	4	8	4	2	4	0.25	0.25	≤0.125	≤0.125	1	0.5	0.25	4	–
*C. freundii*	22	**NDM-5,** CMY-48, CTX-M-15, OXA-1	ND	1	16	≤0.125	64	0.5	≤0.125	8	8	8	2	≤0.125	0.5	0.5	0.25	≤0.125	1	0.25	0.06	2	–
*C. freundii*	ND	**NDM-7**	ND	2	256	≤0.125	>256	4	≤0.125	16	16	64	64	≤0.125	0.25	0.25	≤0.125	≤0.125	2	≤0.125	0.25	1	–
*C. freundii*	ND	**NDM-1,** CTX-M-15	ND	1	256	0.25	>256	8	≤0.125	8	8	16	4	≤0.125	≤0.125	≤0.125	≤0.125	≤0.125	1	≤0.125	0.25	2	–
*C. freundii*	ND	**NDM-1,** CTX-M-15	ND	>256	128	0.125	>256	2	1	4	4	4	1	2	>256	>256	>256	>256	8	>256	0.25	2	–
*E. cloacae*	ND	**NDM-1**	ND	8	128	1	128	8	1	4	4	4	8	4	4	8	4	≤0.125	8	0.25	1	0.5	–
*E. cloacae*	ND	VEB-1	ND	16	32	0.25	4	≤0.125	≤0.125	2	0.25	≤0.25	≤0.125	≤0.125	0.5	0.25	0.5	≤0.125	0.5	1	0.5	2	–
*E. cloacae*	ND	VEB-1	ND	8	64	0.25	2	≤0.125	≤0.125	2	0.25	≤0.25	≤0.125	≤0.125	0.5	0.25	0.5	≤0.125	0.5	1	1	2	–
*E. cloacae*	ND	OXA-48, SHV-12	ND	8	256	1	16	1	≤0.125	2	2	2	1	2	0.25	0.5	0.5	0.5	2	≤0.125	0.25	2	–
*E. cloacae*	66	**NDM-7,** CTX-M-15, TEM-1, OXA-1	ND	1	64	≤0.125	>256	2	≤0.125	8	8	16	8	≤0.125	≤0.125	0.5	≤0.125	≤0.125	2	≤0.125	0.25	2	–
*E. cloacae*	-	**NDM-1,** TEM-1, KLUY-1, OXA-1	N570S	>256	>256	>128	>256	>128	>128	256	>128	>256	64	>128	>128	>128	>128	>128	128	>128	>16	>16	–
*E. cloacae*	ND	**VIM-1,** CTX-M-9	ND	8	>256	≤0.125	>256	16	≤0.125	32	32	32	32	≤0.125	0.25	0.25	0.25	≤0.125	8	0.25	0.25	2	–
*E. cloacae*	ND	**VIM-1,** CTX-M-9	ND	2	256	≤0.125	64	2	≤0.125	16	16	8	4	≤0.125	≤0.125	≤0.125	≤0.125	≤0.125	4	≤0.125	0.25	4	–
*E. cloacae*	ND	**NDM-1,** CTX-M-15	ND	32	256	0.25	>256	8	≤0.125	16	16	64	32	≤0.125	8	16	4	≤0.125	32	≤0.125	0.03	4	–
*E. cloacae*	ND	**NDM-1,** CTX-M-15	ND	64	>256	1	>256	4	≤0.125	8	8	16	16	≤0.125	64	32	16	64	4	64	0.25	2	–
*E. cloacae*	ND	**VIM-1,** CTX-M-15	ND	16	>256	≤0.125	>256	32	≤0.125	16	16	32	16	≤0.125	8	16	1	≤0.125	16	16	0.25	2	–
*E. cloacae*	ND	**VIM-1,** CTX-M-9	ND	4	256	0.5	128	2	≤0.125	4	4	4	4	2	≤0.125	1	≤0.125	≤0.125	4	≤0.125	0.25	1	–
*P. aeruginosa*	235	GES-38	ND	4	64	64	32	32	32	1	1	8	8	16	4	4	8	4	4	8	8	>16	–
*P. aeruginosa*	ND	–	ND	16	64	16	32	32	16	8	2	64	32	64	16	16	16	16	16	16	>16	>16	–
*P. aeruginosa*	ND	VEB-1	ND	16	>256	>256	64	64	64	1	0.5	2	2	1	0.5	1	0.5	0.5	2	2	4	>16	–
*P. aeruginosa*	ND	SHV-12	ND	8	8	4	8	8	8	0.25	0.5	1	2	0.5	0.25	1	1	2	8	1	>16	>16	–
*P. aeruginosa*	ND	VEB-1	ND	8	>256	>256	32	32	32	0.25	≤0.125	0.5	0.5	1	1	2	1	0.5	1	2	2	>16	–
*P. aeruginosa*	357	VIM-5	ND	4	256	256	128	128	>128	256	>128	256	64	>128	2	2	2	4	4	4	0.25	>16	–
*P. aeruginosa*	ND	–	ND	4	128	16	4	4	8	8	4	16	8	16	4	4	8	0.25	8	4	4	>16	–
*P. aeruginosa*	ND	–	ND	8	64	32	8	8	16	16	8	16	16	16	8	8	8	4	8	8	8	>16	–
*P. aeruginosa*	ND	–	ND	8	128	32	32	32	32	16	8	16	16	16	8	8	8	≤0.125	8	16	16	>16	–
*A. baumannii*	85	**NDM-1**	ND	4	>256	>128	>256	128	128	128	128	128	64	128	2	2	2	4	4	2	>16	>16	64
*A. baumannii*	ND	PER-1	ND	64	>256	>128	>256	128	64	0.25	≤0.125	1	0.5	1	0.5	0.5	0.5	0.5	32	2	>16	>16	4
*A. baumannii*	ND	OXA-23, PER-7	ND	256	>256	>128	256	64	32	64	64	32	32	64	2	≤0.125	1	0.5	1	2	>16	>16	0.5
*A. baumannii*	ND	OXA-23, PER-7	ND	128	>256	>128	256	32	32	64	64	16	32	64	1	≤0.125	0.5	0.5	2	1	>16	>16	0.5
*A. baumannii*	ND	OXA-23, PER-1	ND	64	>256	>128	256	64	32	32	32	32	32	32	0.5	0.5	0.5	0.5	1	1	>16	>16	1
*A. baumannii*	ND	OXA-23, PER-1	ND	128	>256	>128	256	128	32	64	64	64	64	64	0.5	0.25	0.25	0.5	2	1	>16	>16	0.5
*A. baumannii*	ND	OXA-23, PER-7	ND	128	>256	>128	>256	32	32	32	32	16	32	32	1	0.5	0.5	0.5	2	2	>16	>16	0.25
*A. baumannii*	ND	OXA-23, PER-7	ND	32	>256	>128	256	32	64	32	32	16	32	32	1	0.25	0.5	0.5	2	1	>16	>16	2
*A. baumannii*	ND	OXA-23, PER-7	ND	128	>256	>128	256	64	32	32	32	64	32	64	0.5	0.5	0.25	0.5	2	2	>16	>16	1
*A. baumannii*	ND	**NDM-5**, OXA-23	ND	1	>256	>128	>256	>128	>128	256	>128	256	64	>128	1	0.5	1	2	1	2	>16	>16	128
*A. baumannii*	ND	**NDM-1**, OXA-23	ND	4	128	>128	>256	>128	>128	256	>128	>256	64	>128	2	1	2	2	4	4	>16	>16	128

^a^Antibiotic abbreviations: *ATM* aztreonam, *FDC* cefiderocol, *AZA* aztreonam/avibactam, *FEP* cefepime, *FEP-TAN* cefepime/taniborbactam, *FEP-ZID* cefepime/zidebactam, *IPM *imipenem, *I/R* imipenem/relebactam, *MEM* meropenem, *MEM-NAC* meropenem/nacubactam, *FDC-AVI* cefiderocol/avibactam, *FDC-REL* cefiderocol/relebactam, *FDC-TAN* cefiderocol/taniborbactam, *FDC-ZID* cefiderocol/zidebactam, *FDC-NAC* cefiderocol/nacubactam, SUL-DUR sulbactam/durlobactam; the concentration of β-lactamase inhibitors were fixed at 4 mg/L for avibactam, relebactam, taniborbactam, zidebactam, nacubactam and durlobactam. The concentration of vaborbactam was fixed at 8mg/L

^b^MBL enzymes are boldened

^c^*ND*, data not available

### Susceptibility testing

MIC values were determined in duplicate by broth microdilution method using Mueller-Hinton (Bio-Rad Laboratories, Hercules, USA) for all β-lactams and their combinations except for cefiderocol, for which an iron-depleted Mueller-Hinton was used in accordance with EUCAST guidelines [29]. MIC value of the ongoing BL/BLI, cefepime-taniborbactam, cefepime-zidebactam, meropenem-nacubactam, and sulbactam-durlobactam was determined using a fixed concentration at 4 mg/L for these inhibitors [17, 26, 30]. The clinically used BL/BLI combinations aztreonam-avibactam, meropenem-vaborbactam, and imipenem-relebactam were also evaluated for comparison using a fixed concentration of 4 mg/L for avibactam and relebactam, and 8 mg/L for vaborbactam. Susceptibility of cefiderocol was determined alone or in combination with a fixed concentration of avibactam (4 mg/L) (cefiderocol-avibactam), relebactam (4 mg/L) (cefiderocol-relebactam), taniborbactam (4 mg/L) (cefiderocol-taniborbactam), zidebactam (4 mg/L) (cefiderocol-zidebactam), vaborbactam (8mg/L) (cefiderocol-vaborbactam), and nacubactam (4 mg/L) (cefiderocol-nacubactam) [30]. Interpretation was based on EUCAST breakpoints if available; otherwise, the breakpoints were chosen according to the corresponding β-lactam included in the BL/BLI combination [29]. Hence, resistance to cefepime-taniborbactam, cefepime-zidebactam, and meropenem-nacubactam (meropenem-nacubactam) were defined as MIC values > 4 mg/L for Enterobacterales and > 8 mg/L for *P. aeruginosa* and *A. baumannii*, whereas susceptibility was defined as MIC values ≤ 1 mg/L for cefepime-based combinations and ≤ 2 mg/L for meropenem-nacubactam for Enterobacterales, and MIC values ≤ 8 mg/L for cefepime-taniborbactam, cefepime-zidebactam, and meropenem-nacubactam for *P. aeruginosa* and *A. baumannii*. Resistance to sulbactam-durlobactam combination was defined as MIC values > 4 mg/L in *A. baumannii*, and resistance to cefiderocol and cefiderocol-based combinations were defined as MIC values > 2 mg/L for Enterobacterales and *P. aeruginosa.* In order to further evaluate the contribution of zidebactam and nacubactam that possess significant antibacterial activity on their own [17, 30], MICs of zidebactam and nacubactam alone were also determined. To better describe our strain collection, not only MIC_50_ but also MIC_90_ values of all β-lactams were determined and provided here.

## Results

### Susceptibility to the newly developed BL/BLI combinations in Enterobacterales

All enterobacterial clinical isolates tested included in our study showed resistance or reduced susceptibility to cefiderocol. Of note, 38.8% of those isolates showed an MIC value of cefiderocol close to the susceptibility breakpoint, being at 1 or 2 mg/L. The cefepime-zidebactam combination was the most effective with 91% of isolates showing MIC values ≤ 1 mg/L, and MIC_50_ and MIC_90_ values being at 0.125 mg/L and 1 mg/L, respectively (Table 2). The second-best option was meropenem-nacubactam with 71.6% of isolates exhibiting MIC values ≤ 2 mg/L, and MIC_50_ and MIC_90_ values being respectively at 0.125 mg/L and 128 mg/L. Of note, these results were mainly related to the MIC_50_ and MIC_90_ values for zidebactam and nacubactam alone evaluated at 0.125 and 2 mg/L and 2 and 8 mg/L, respectively, thus to the intrinsic activity of those latter molecules as antibacterial agents rather to their capacity to inhibit the corresponding β-lactamase genes. Interestingly, these two combinations were more effective than aztreonam-avibactam and cefepime-taniborbactam, which, along with cefiderocol, are considered last-line options against MBL producers. Given the following susceptibility rates obtained, respectively, 37.3% for meropenem-vaborbactam and 10.4% for imipenem-relebactam, those combinations did not display a high effectiveness against this strain collection. All these data are consistent with the results provided according to the production of NDM-like or VIM-like enzymes (Table S1). The activity of the combinations mentioned above was overall the same when testing PBP3-modified *E. coli*, therefore evidencing that this latter feature was not playing a major role in the resistance phenotype for those combinations. Nevertheless, it is worth highlighting that the percentage of enterobacterial isolates displaying MIC values ≤ 1 mg/L for cefepime-taniborbactam was only 19.4%, and was 0% among the PBP3-modified *E. coli* strains, suggesting a cross-resistance between cefiderocol and cefepime-taniborbactam.

**Table 2:** Cumulative MIC distribution of novel BL/BLI commercially-available or under clinical development based on aztreonam, cefepime, imipenem, and meropenem for 67 cefiderocol-reduced susceptibility/resistant *Enterobacterales* strains, 9 cefiderocol-resistant *Pseudomonas aeruginosa* strains, and 11 cefiderocol-resistant *Acinetobacter baumannii*

Isolate type	Cumulative % of isolate at MIC (mg/L)												% of susceptible isolates^b^	MIC_50_	MIC_90_	Range
	BL/BLI combinations^a^	≤0.125	0.25	0.5	1	2	4	8	16	32	64	≥128				
Enterobacterales *n* = 67	FDC				17.9	38.8	50.7	67.2	76.1	88.1	92.5	100	38.8	4	32	≤0.25 >256
	ATM		3	6	6	7.5	7.5	7.5	10.4	14.9	29.9	100	6	256	256	≤0.25 >256
	AZA	34.3	44.8	53.7	65.7	76.1	79.1	83.6	89.6	92.5	92.5	100	65.7	0.5	32	≤0.125 >128
	FEP					1.5	3	4.5	6	10.4	14.9	100	0	256	256	≤0.25 >256
	FEP-TAN	4.5	6	13.4	19.4	28.4	34.3	40.3	50.7	64.2	85.1	100	19.4	16	128	≤0.125 >128
	FEP-ZID	82.1	86.6	86.6	91	91	91	92.5	92.5	92.5	92.5	100	91	0.125	1	≤0.125 >128
	IPM			1.5	4.5	10.4	23.9	50.7	71.6	82.1	91	100	10.4	8	64	≤0.25 >256
	I/R	1.5	6	6	6	10.4	26.9	52.2	71.6	82.1	94	100	10.4	8	64	≤0.125 >128
	MEM		3	3	6	10.4	20.9	29.9	43.3	56.7	77.6	100	10.4	32	128	≤0.25 >256
	MVB	4.5	4.5	4.5	10.4	17.9	28.4	37.3	52.2	74.6	100	100	37.3	16	64	≤0.125 >128
	MER-NAC	56.7	58.2	61.2	61.2	71.6	80.6	80.6	80.6	82.1	89.6	100	71.6	0.125	128	≤0.125 >128
PBP3 modified *E. coli n*= 25	FDC					20	28	40	60	80	88	100	20	16	128	≤0.25 >256
	ATM			8	8	12	12	12	16	16	32	100	8	256	256	≤0.25 >256
	AZA	8	8	20	44	64	72	84	96	100	100	100	44	2	16	≤0.125 >128
	FEP							4	4	4	4	100	0	256	256	≤0.25 >256
	FEP-TAN							4	16	40	88	100	0	64	128	≤0.125 >128
	FEP-ZID	100	100	100	100	100	100	100	100	100	100	100	100	0.125	0.125	≤0.125 >128
	IPM			4	4	4	8	44	80	96	100	100	4	16	32	≤0.25 >256
	I/R		4	4	4	4	8	44	76	96	100	100	4	16	32	≤0.125 >128
	MEM				4	4	4	4	24	44	88	100	4	64	128	≤0.25 >256
	MVB	4	4	4	4	4	4	12	44	84	100	100	12	32	64	≤0.125 >128
	MER-NAC	64	64	68	68	68	72	72	72	76	92	100	68	0.125	64	≤0.125 >128
*Pseudomonas aeruginosa* *n* = 9	FDC						33	77.8	100	100	100	100	0	8	16	≤0.25 >256
	ATM							11.1	11.1	11.1	55.6	100	11.1	64	256	≤0.25 >256
	AZA						11.1	11.1	33.3	44.4	66.7	100	33.3	64	128	≤0.125 >128
	FEP						11.1	11.1	33.3	55.6	66.7	100	11.1	32	256	≤0.25 >256
	FEP-TAN						11.1	33.3	33.3	77.8	88.9	100	33.3	32	128	≤0.125 >128
	FEP-ZID							22.2	44.4	77.8	88.9	100	22.2	32	128	≤0.125 >128
	IPM		22.2	22.2	44.4	44.4	44.4	66.7	88.9	88.9	88.9	100	44.4	8	64	≤0.25 >256
	I/R	11.1	11.1	33.3	44.4	55.6	66.7	88.9	88.9	88.9	88.9	100	55.6	2	32	≤0.125 >128
	MEM			11.1	22.2	33.3	33.3	44.4	77.8	77.8	88.9	100	33.3	16	128	≤0.25 >256
	MVB			11.1	22.2	33.3	33.3	55.6	77.8	88.9	100	100	55.6	8	64	≤0.125 >128
	MER-NAC			11.1	33.3	33.3	33.3	33.3	77.8	77.8	88.9	100	33.3	16	128	≤0.125 >128
*Acinetobacter baumannii* *n* = 11	FDC				9.1	9.1	27.3	27.3	27.3	36.4	54.5	100	9.1	64	128	≤0.25 >256
	ATM											100	0	256	256	≤0.25 >256
	AZA											100	0	128	128	≤0.125 >128
	FEP											100	0	256	256	≤0.25 >256
	FEP-TAN									27.3	54.5	100	0	64	128	≤0.125 >128
	FEP-ZID									54.5	72.7	100	0	32	128	≤0.125 >128
	IPM		9.1	9.1	9.1	9.1	9.1	9.1	9.1	45.5	72.7	100	9.1	64	256	≤0.25 >256
	I/R		9.1	9.1	9.1	9.1	9.1	9.1	9.1	45.5	72.7	100	9.1	64	128	≤0.125 >128
	MEM				9.1	9.1	9.1	9.1	36.4	54.5	72.7	100	9.1	32	256	≤0.25 >256
	MVB			9.1	9.1	9.1	9.1	9.1	9.1	63.6	100	100	9.1	32	64	≤0.125 >128
	MER-NAC				9.1	9.1	9.1	9.1	36.4	54.5	72.7	100	9.1	64	128	≤0.125 >128
	SUL-DUR		9.1	36.4	54.5	63.6	72.7	72.7	72.7	72.7	81.8	100	72.7	1	128	≤0.125 >128

^a^Antibiotic abbreviations: *ATM* aztreonam, *FDC* cefiderocol, *AZA* aztreonam/avibactam, *FEP* cefepime, *FEP-TAN *cefepime/taniborbactam, *FEP-ZID *cefepime/zidebactam, *IPM *imipenem, *I/R* imipenem/relebactam, *MEM* meropenem, *MVB* meropenem/vaborbactam, *MEM-NAC* meropenem/nacubactam, *SUL-DUR* sulbactam-durlobactam; the concentration of β-lactamase inhibitors were fixed at 4mg/L for avibactam, relebactam, taniborbactam, zidebactam, nacubactam, and durlobactam. The concentration of vaborbactam was fixed at 8mg/L

^b^According to EUCAST, if the breakpoint is available, otherwise according to the respective β-lactam in the β-lactam/β-lactamase inhibitor combination; for *P. aeruginosa*, due to the very low MIC susceptible breakpoints for cefepime, imipenem, and aztreonam according to EUCAST, all isolates with MIC values lower than the resistant breakpoint value for those antibiotics and their combinations were considered susceptible strains

### Susceptibility to cefiderocol in combination with β-lactamase inhibitors in Enterobacterales

When testing Enterobacterales isolates, all combinations of cefiderocol and β-lactamase inhibitors exhibited higher susceptibility levels (lower MICs) than for cefiderocol alone, as shown in Table 3. Considering an MIC breakpoint at 2 mg/L, the susceptibility rates were highest for cefepime-zidebactam (91%), followed by cefiderocol-taniborbactam (76.1%), cefiderocol-nacubactam (74.6%), cefiderocol-avibactam (64.7%), cefiderocol-relebactam (59.7%), and cefiderocol-vaborbactam (22.4%). Of note, lower susceptibility rates were found when testing the PBP3-modified *E. coli* strains, likely due to the contribution of these modifications in the reduced susceptibility to cefiderocol. For those latter mutated isolates, the best combinations were cefiderocol-zidebactam (88%) and cefiderocol-nacubactam (64%), likely due to the high bactericidal activity of these both BLIs in this specie. All these data are in line with the results obtained after classification of NDM-like or VIM-like producers (Table S1).

**Table 3:** Cumulative MIC distribution of cefiderocol in the presence or absence of avibactam, relebactam, vaborbactam, zidebactam, taniborbactam, and nacubactam for 67 cefiderocol-reduced susceptibility *Enterobacterales* strains, 9 cefiderocol-resistant *Pseudomonas aeruginosa* strains, and 11 cefiderocol-resistant *Acinetobacter baumannii* strains

Isolate type		Cumulative % of isolate at MIC (mg/L)											% of susceptible isolates^b^	MIC_50_	MIC_90_	Range
	BL/BLI combinations^a^	≤0.125	0.25	0.5	1	2	4	8	16	32	64	≥128				
Enterobacterales (All) *n* = 67	FDC				17.9	38.8	50.7	67.2	76.1	88.1	92.5	100	38.8	4	32	≤0.25 >256
	FDC-AVI	28.4	40.3	49.3	59.7	64.7	72.1	76.5	86.8	89.7	92.6	100	64.7	1	32	≤0.125 >128
	FDC-REL	17.9	32.8	44.8	53.7	59.7	61.2	71.6	83.6	91	92.5	100	59.7	1	32	≤0.125 >128
	FDC-VAB			3	22.4	46.3	58.2	74.6	83.6	92.5	92.5	100	22.4	4	32	≤0.125 >128
	FDC-TAN	26.9	47.8	56.7	71.6	76.1	83.6	88.1	92.5	95.5	95.5	100	76.1	0.5	16	≤0.125 >128
	FDC-ZID	79.1	82.1	88.1	91	91	92.5	92.5	94	95.5	95.5	100	91	0.125	1	≤0.125 >128
	FDC-NAC	46.3	58.2	64.2	74.6	79.1	83.6	86.6	92.5	94	95.5	100	74.6	0.25	16	≤0.125 >128
PBP3 modification *n*= 25	FDC					20	28	40	60	80	88	100	20	16	128	≤0.25 >256
	FDC-AVI	4	12	16	20	32	44	52	76	84	88	100	32	8	128	≤0.125 >128
	FDC-REL			12	20	28	32	48	68	84	88	100	28	8	128	≤0.125 >128
	FDC-VAB				4	24	28	48	68	84	84	100	24	16	128	≤0.125 >128
	FDC-TAN	4	20	24	48	56	68	80	88	96	96	100	56	2	32	≤0.125 >128
	FDC-ZID	72	80	88	88	88	92	92	92	96	96	100	88	0.125	4	≤0.125 >128
	FDC-NAC	28	40	40	52	64	76	80	92	96	96	100	64	1	16	≤0.125 >128
*Pseudomonas aeruginosa* *n* = 9	FDC						33	77.8	100	100	100	100	0	8	16	≤0.25 >256
	FDC-AVI		11.1	22.2	33.3	44.4	66.7	88.9	100	100	100	100	44.4	4	16	≤0.125 >128
	FDC-REL				22.2	44.4	66.7	88.9	100	100	100	100	44.4	4	16	≤0.125 >128
	FDC-VAB				11.1	22.2	44.4	88.9	100	100	100	100	22.2	8	16	≤0.125 >128
	FDC-TAN			11.1	33.3	44.4	44.4	88.9	100	100	100	100	44.4	8	16	≤0.125 >128
	FDC-ZID	11.1	22.2	44.4	44.4	55.6	88.9	88.9	100	100	100	100	55.6	2	8	≤0.125 >128
	FDC-NAC				11.1	33.3	55.6	77.8	100	100	100	100	33.3	4	16	≤0.125 >128
*Acinetobacter baumanii* *n* = 11	FDC				9.1	9.1	27.3	27.3	27.3	36.4	54.5	100	9.1	64	128	≤0.25 >256
	FDC-AVI			36.4	72.7	100	100	100	100	100	100	100	100	1	2	≤0.125 >128
	FDC-REL	18.2	36.4	81.8	90.9	90.9	100	100	100	100	100	100	90.9	0.5	2	≤0.125 >128
	FDC-VAB				27.7	72.7	90.9	90.9	90.9	100	100	100	72.7	2	4	≤0.125 >128
	FDC-TAN		18.2	63.6	81.8	100	100	100	100	100	100	100	100	0.5	2	≤0.125 >128
	FDC-ZID			72.7	81.8	90.9	100	100	100	100	100	100	90.9	0.5	2	≤0.125 >128
	FDC-NAC				45.5	81.8	100	100	100	100	100	100	81.8	2	2	≤0.125 >128

^a^Antibiotic abbreviations: *FDC* cefiderocol, *FDC-AVI* cefiderocol/avibactam, *FDC-REL* cefiderocol/relebactam, *FDC-VAB* cefiderocol/vaborbactam, *FDC-TAN* cefiderocol/taniborbactam, *FDC-ZID* cefiderocol/zidebactam, *FDC-NAC* cefiderocol/nacubactam; the concentration of β-lactamase inhibitors were fixed at 4 mg/L for avibactam, relebactam, taniborbactam, zidebactam, and nacubactam. The concentration of vaborbactam was fixed at 8mg/L

^b^According to cefiderocol EUCAST breakpoint defining susceptible strains as MIC value ≤ 2 mg/L for Enterobacterales, and *P. aeruginosa.* Breakpoint defined as MIC value ≤ 2 mg/L for *A. baumannii*

### Susceptibility to BL/BLI and cefiderocol/inhibitor combinations for *P. aeruginosa* and *A. baumannii*

For *P. aeruginosa*, the combinations that showed the higher susceptibility rates were imipenem-relebactam and meropenem-vaborbactam, with 55.6% of the isolates being susceptible. On the other hand, the following combinations aztreonam-avibactam, cefepime-taniborbactam, cefepime-zidebactam, and meropenem-nacubactam showed poor activities (MIC_50_ ≥ 16 mg/L) against those *P. aeruginosa* isolates showing reduced susceptibility to cefiderocol. Worryingly, a poor activity of those BL/BLI combinations was also observed with *A. baumannii* (all MIC_50_ ≥ 32 mg/L)*.* The only effective combo against those *A. baumannii* isolates was SUL-DUR as 72.7% of isolates had MIC values ≤ 4 mg/L. MIC results for all clinical isolates showing reduced susceptibility or resistance to cefiderocol are shown in Table 1, including their β-lactam resistance determinants. Regarding cefiderocol combinations, the addition of β-lactamase inhibitors contributed to significantly decrease the MIC values for *P. aeruginosa*. Hence, cefiderocol-zidebactam showed the highest susceptibility rate (55.6%), whereas it was only 44.4% or less for all other combinations, the lowest rate being observed with cefiderocol-vaborbactam reporting (22.2%). For *A. baumannii*, combinations including cefiderocol with either avibactam, zidebactam, tanoborbactam, or relebactam displayed susceptibility rates higher than 90%, although these rates were at 9.1% for cefiderocol alone.

## Discussion

Our data highlighted that the best in vitro activities against Enterobacterales showing reduced susceptibility or resistance to cefiderocol are cefepime-zidebactam and meropenem-nacubactam, even against *E. coli* strains exhibiting PBP3 modifications*.* In addition, MIC values of cefiderocol combined with BLIs, especially zidebactam, nacubactam, and taniborbactam, were significantly lower than those of cefiderocol alone. Those data might be explained by several reasons. First, the reduced susceptibility to cefiderocol in Enterobacterales was mainly associated to the co-production of CMY-like and/or SHV-like β-lactamases in addition to NDM-like enzymes, the former having been already shown to contribute to cefiderocol resistance [8]. Hence, combining cefiderocol with a BLI that can antagonize the activity of those CMY- or SHV-type β-lactamases basically constitute a significant advantage [8, 9]. Second, zidebactam and nacubactam have not only the ability to inhibit class A β-lactamases, but also possess a significant antibacterial activity by targeting the PBP-2 of Enterobacterales; therefore, those BLIs exhibit a so-called enhancer activity, in line with the low MIC values observed in this study and another [30]. Third, our collection included a high proportion of PBP3-modified *E. coli* strains, that latter feature being known to affect the susceptibility to cefiderocol, but also that of aztreonam-avibactam and cefepime-taniborbactam, considering that cefiderocol, aztreonam, and cefepime mainly act by targeting the PBP-3 [22, 31–33]. Of note, imipenem-relebactam and meropenem-vaborbactam did not show any significant antibacterial activity against the Enterobacterales tested, which can be explained by the majority being MBL producers.

When considering cefiderocol-resistant *P. aeruginosa* isolates, the most efficient option was imipenem-relebactam, likely due a high proportion of AmpC overproducers and/or co-producers of SHV-like or VEB-like ESBLs. These results are in line with other studies reporting that the imipenem-relebactam combination is one of the best alternatives to treat infections caused by imipenem-non susceptible *P. aeruginosa*, mainly driven by multiple resistances mechanisms including combinations of OprD inactivation and AmpC or/and efflux overexproduction [34]. Noteworthy, avibactam and vaborbactam have a negligible effect on restoring susceptibility to aztreonam and meropenem, respectively, probably related to the fact that both β-lactams are very good substrates of the MexAB-OprM efflux system, which is commonly overexpressed in a large proportion of clinical isolates and against which the addition of β-lactamase inhibitors is ineffective. Interestingly, the β-lactamase inhibitor relebactam not only showed an excellent inhibitory activity against class A β-lactamases (i.e., SHV-like and VEB-like enzymes), but also against class C β-lactamases such as the intrinsic PDC-like enzymes. Hence, the association of relebactam with imipenem could restore low MIC values against isogenic imipenem non-susceptible *P. aeruginosa* isolates producing a wide range of non-enzymatic resistance mechanisms identified in that species, eventually contributing to cefiderocol resistance [34, 35]. On the other hand, the cefepime-taniborbactam and cefepime-zidebactam combinations were not as effective against *P. aeruginosa* isolates as they were against Enterobacterales. This may be explained by the fact that cefepime is particularly affected by the overexproduction of efflux pumps such as MexAB-OprM, or MexXY. In addition, we did not analyze the presence or absence of *bla*_OXA-like_ in our *P. aeruginosa* isolate collection, and it may be that zidebactam and taniborbactam are not efficient β-lactamase inhibitors against OXA-2- or OXA-10-type β-lactamases, which are known to be widely distributed in *P. aeruginosa* and contributing to the reduced susceptibility to cefiderocol [11, 19]. Finally, zidebactam and nacubactam have a lower intrinsic antibacterial activity against *P. aeruginosa* in comparison to Enterobacterales, considering that the MIC values are approximately around 4–16 mg/L for zidebactam, and above 32 mg/L for nacubactam for *Pseudomonas* spp. [36, 37].

When considering cefiderocol-resistant *A. baumannii*, the main resistance mechanism is driven by the production of PER-like or NDM-like enzymes, as already described [9]. Our data showed that aztreonam-avibactam was not an interesting option, since *A. baumannii* naturally exhibits high MIC values of aztreonam, and this β-lactam is also hydrolyzed at high level by PER-like enzymes, often produced in that species [9, 17]. Additionally, the class D β-lactamase OXA-23 (which is very commonly produced in carbapenem-resistant isolates) significantly hydrolyzes imipenem and meropenem, but neither nacubactam nor relebactam inhibit OXA-23-like enzymes, eventually leading to resistance to meropenem-nacubactam and imipenem-relebactam. However, durlobactam inhibits class A β-lactamases, such as PER-like enzymes, as well as some class D β-lactamases such as OXA-23, which explains the high susceptibility rate observed for sulbactam-durlobactam in this strain collection. Cefepime being a very good substrate for PER-like enzyme, but also for efflux pumps oftenly overproduced in *A. baumannii*, this may explain why combinations including taniborbactam and zidebactam were not sufficiently active to restore MIC values in the susceptibility range. Finally, it is worth highlighting that zidebactam and nacubactam do not possess significant direct antibacterial activity in that species [36, 37]. The main feature to be highlighted is that combining cefiderocol with most β-lactamase inhibitors in this study resulted in low MIC values for most isolates. This is most likely due to the ability of all BLI tested to inhibit PER-like enzymes, which were the most relevant resistance factors of cefiderocol detected in this specie. Those results strongly suggest that combination therapies including cefiderocol with a β-lactamase inhibitor, and sulbactam-durlobactam, might be extremely effective in the treatment of infections caused by cefiderocol-resistant *A. baumannii* isolates.

In conclusion, we showed here that cefepime-zidebactam, meropenem-nacubactam, and aztreonam-avibactam are the best therapeutic alternatives against multidrug-resistant Enterobacterales exhibiting reduced susceptibility or resistance to cefiderocol. By contrast, imipenem-relebactam was the best option against *P. aeruginosa* isolates*.* Worryingly, with the exception of sulbactam-durlobactam, none of the novel β-lactam/β-lactamase inhibitor combinations were effective against *A. baumannii* isolates. Nevertheless, combinations made of cefiderocol and several β-lactamase inhibitors (namely avibactam, taniborbactam, relebectam, zidebactam, and nacubactam) constitute interesting alternative therapeutics against all tested Gram-negative clinical isolates showing reduced susceptibility to cefiderocol.

### Supplementary information

ESM 1 (DOCX 35 kb)

## Data Availability

All data generated through this study can be available upon request.

## References

[1] Nordmann P, Poirel L (2019). Epidemiology and diagnostics of carbapenem resistance in Gram-negative bacteria. Clin Infect Dis.

[2] World Health Organization. Global priority list of antibiotic-resistant bacteria to guide research, discovery, and development of new antibiotics. 2017 Available at: "http://www.who.int/medicines/publications/WHO-PPL-Short_Summary_25Feb-ET_NM_WHO.pdf.%20Accessed%2024%20July%202023"

[3] Quale J, Bratu S, Gupta J, Landman D (2006). Interplay of efflux system, ampC, and oprD expression in carbapenem resistance of *Pseudomonas aeruginosa* clinical isolates. Antimicrob Agents Chemother.

[4] Rodríguez-Martínez JM, Poirel L, Nordmann P (2009). Molecular epidemiology and mechanisms of carbapenem resistance in *Pseudomonas aeruginosa*. Antimicrob Agents Chemother.

[5] Nikaido H (2003). Molecular basis of bacterial outer membrane permeability revisited. Microbiol Mol Biol Rev.

[6] Hsu LY, Apisarnthanarak A, Khan E, Suwantarat N, Ghafur A, Tambyah PA (2017). Carbapenem-resistant *Acinetobacter baumannii* and Enterobacteriaceae in South and Southeast Asia. Clin Microbiol Rev.

[7] Tamma PD, Aitken SL, Bonomo RA, Mathers AJ, van Duin D, Clancy CJ (2022). Infectious Diseases Society of America 2022 Guidance on the treatment of extended-spectrum β-lactamase producing Enterobacterales (ESBL-E), carbapenem-resistant Enterobacterales (CRE), and Pseudomonas aeruginosa with difficult-to-treat resistance (DTR-P. aeruginosa). Clin Infect Dis.

[8] Poirel L, Ortiz de la Rosa JM, Sadek M, Nordmann P (2022). Impact of acquired broad-spectrum β-lactamases on susceptibility to cefiderocol and newly developed β-lactam/β-lactamase inhibitor combinations in *Escherichia coli* and *Pseudomonas aeruginosa*. Antimicrob Agents Chemother.

[9] Poirel L, Sadek M, Nordmann P (2021). Contribution of PER-type and NDM-type β-lactamases to cefiderocol resistance in *Acinetobacter baumannii*. Antimicrob Agents Chemother.

[10] Poirel L, Sadek M, Kusaksizoglu A, Nordmann P (2022). Co-resistance to ceftazidime-avibactam and cefiderocol in clinical isolates producing KPC variants. Eur J Clin Microbiol Infect Dis.

[11] Vuillemin X, Da Silva M, Bour M, Landon C, Plésiat P, Jeannot K (2023). cefiderocol activity is compromised by acquired Extended Spectrum-Oxacillinases in *Pseudomonas aeruginosa*. Int J Antimicrob Agents.

[12] Sadek M, Le Guern R, Kipnis E, Gosset P, Poirel L, Dessein R, Nordmann P (2023). Progressive in vivo development of resistance to cefiderocol in *Pseudomonas aeruginosa*. Eur J Clin Microbiol Infect Dis.

[13] Takemura M, Yamano Y, Matsunaga Y, Ariyasu M, Echols R, Nagata TD (2020). 1266. Characterization of shifts in minimum inhibitory concentrations during treatment with cefiderocol or comparators in the phase 3 CREDIBLE-CR and APEKS-NP studies. Open Forum. Infect Dis.

[14] Yahav D, Giske CG, Grāmatniece A, Abodakpi H, Tam VH, Leibovici L (2020). New β-lactam-β-lactamase inhibitor combinations. Clin Microbiol Rev.

[15] Le Terrier C, Gruenig V, Fournier C, Nordmann P, Poirel L (2023). NDM-9 resistance to taniborbactam. Lancet Infect Dis.

[16] Le Terrier C, Nordmann P, Buchs C, Di DYW, Rossolini GM, Stephan R, Castanheira M, Poirel L (2023). Wide dissemination of Gram-negative bacteria producing the taniborbactam-resistant NDM-9 variant: a One Health concern. J Antimicrob Chemother.

[17] Le Terrier C, Nordmann P, Freret C, Seigneur M, Poirel L (2023). Impact of acquired broad spectrum β-lactamases on susceptibility to novel combinations made of β-lactams (aztreonam, cefepime, meropenem, and imipenem) and novel β-lactamase inhibitors in Escherichia coli and Pseudomonas aeruginosa. Antimicrob Agents Chemother.

[18] Le Terrier C, Viguier C, Nordmann P, Poirel L (2023). Relative inhibitory activities of the broad-spectrum ß-lactamase inhibitor taniborbactam against metallo-ß-lactamases.

[19] Lasarte-Monterrubio C, Fraile-Ribot PA, Vázquez-Ucha JC, Cabot G, Guijarro-Sánchez P, Alonso-García I, Rumbo-Feal S, Galán-Sánchez F, Beceiro A, Arca-Suárez J, Oliver A, Bou G (2022). Activity of cefiderocol, imipenem/relebactam, cefepime/taniborbactam and cefepime/zidebactam against ceftolozane/tazobactam- and ceftazidime/avibactam-resistant *Pseudomonas aeruginosa*. J Antimicrob Chemother.

[20] Vázquez-Ucha JC, Lasarte-Monterrubio C, Guijarro-Sánchez P, Oviaño M, Álvarez-Fraga L, Alonso-García I, Arca-Suárez J, Bou G, Beceiro A, GEMARA-SEIMC/REIPI Enterobacterales Study Group (2022). Assessment of activity and resistance mechanisms to cefepime in combination with the novel β-lactamase inhibitors zidebactam, taniborbactam, and enmetazobactam against a multicenter collection of carbapenemase-producing Enterobacterales. Antimicrob Agents Chemother.

[21] Karlowsky JA, Hackel MA, Wise MG, Six DA, Uehara T, Daigle DM, Cusick SM, Pevear DC, Moeck G, Sahm DF (2023). In vitro activity of cefepime-taniborbactam and comparators against clinical isolates of Gram-negative bacilli from 2018 to 2020: results from the Global Evaluation of Antimicrobial Resistance via Surveillance (GEARS) Program. Antimicrob Agents Chemother.

[22] Le Terrier C, Nordmann P, Sadek M, Poirel L (2023). In vitro activity of cefepime/zidebactam and cefepime/taniborbactam against aztreonam/avibactam-resistant NDM-like-producing *Escherichia coli* clinical isolates. J Antimicrob Chemother.

[23] ClinicalTrials.gov (2022). Study of cefepime-zidebactam (FEP-ZID) in complicated urinary tract infection (cUTI) or acute pyelonephritis (AP). https://clinicaltrials.gov/ct2/show/NCT04979806. Accessed 1 Nov 2023

[24] ClinicalTrials.gov (2022). Safety and efficacy study of cefepime/VNRX-5133 in patients with complicated urinary tract infections (CERTAIN-1). https://clinicaltrials.gov/ct2/show/NCT03840148. Accessed 1 Nov 2023

[25] ClinicalTrials.gov (2022). A study to investigate the intrapulmonary lung penetration of nacubactam in healthy participants. https://clinicaltrials.gov/ct2/show/NCT03182504. Accessed 1 Nov 2023

[26] Findlay J, Poirel L, Bouvier M, Nordmann P (2022). In vitro activity of sulbactam-durlobactam against carbapenem-resistant Acinetobacter baumannii and mechanisms of resistance. J Glob Antimicrob Resist.

[27] Kaye KS, Shorr AF, Wunderink RG, Du B, Poirier GE, Rana K, Miller A, Lewis D, O'Donnell J, Chen L, Reinhart H, Srinivasan S, Isaacs R, Altarac D (2023). Efficacy and safety of sulbactam-durlobactam versus colistin for the treatment of patients with serious infections caused by *Acinetobacter baumannii-calcoaceticus* complex: a multicentre, randomised, active-controlled, phase 3, non-inferiority clinical trial (ATTACK). Lancet Infect Dis.

[28] Hackel MA, Tsuji M, Yamano Y, Echols R, Karlowsky JA, Sahm DF (2019). Reproducibility of broth microdilution MICs for the novel siderophore cephalosporin, cefiderocol, determined using iron-depleted cation-adjusted Mueller-Hinton broth. Diagn Microbiol Infect Dis.

[29] EUCAST (2023) Breakpoint tables for interpretation of MICs and zone diameters. Version 13:1. https://www.eucast.org/fileadmin/src/media/PDFs/EUCAST_files/Breakpoint_tables/v_13.1_Breakpoint_Tables.pdf. Accessed 1 Nov 2023

[30] Le Terrier C, Nordmann P, Poirel L (2022). In vitro activity of aztreonam in combination with newly developed β-lactamase inhibitors against MDR Enterobacterales and *Pseudomonas aeruginosa* producing metallo-β-lactamases. J Antimicrob Chemother.

[31] Sadek M, Juhas M, Poirel L, Nordmann P (2020). Genetic features leading to reduced susceptibility to aztreonam-avibactam among metallo-β-lactamase-producing *Escherichia coli* isolates. Antimicrob Agents Chemother.

[32] Sato T, Ito A, Ishioka Y, Matsumoto S, Rokushima M, Kazmierczak KM, Hackel M, Sahm DF, Yamano Y (2020). Escherichia coli strains possessing a four amino acid YRIN insertion in PBP3 identified as part of the SIDERO-WT-2014 surveillance study. JAC Antimicrob Resist.

[33] Davies TA, Shang W, Bush K, Flamm RK (2008). Affinity of doripenem and comparators to penicillin-binding proteins in *Escherichia coli* and *Pseudomonas aeruginosa*. Antimicrob Agents Chemother.

[34] Fraile-Ribot PA, Zamorano L, Orellana R, Del Barrio-Tofiño E, Sánchez-Diener I, Cortes-Lara S, López-Causapé C, Cabot G, Bou G, Martínez-Martínez L, Oliver A, GEMARA-SEIMC/REIPI Pseudomonas Study Group (2020). Activity of imipenem-relebactam against a large collection of *Pseudomonas aeruginosa* clinical isolates and isogenic β-lactam-resistant mutants. Antimicrob Agents Chemother.

[35] Hilbert DW, DeRyke CA, Motyl M, Hackel M, Young K (2023). Relebactam restores susceptibility of resistant *Pseudomonas aeruginosa* and Enterobacterales and enhances imipenem activity against chromosomal AmpC-producing species: analysis of global SMART 2018-2020. BMC Microbiol.

[36] Livermore DM, Mushtaq S, Warner M, Woodford N (2015). Activity of OP0595/β-lactam combinations against Gram-negative bacteria with extended-spectrum, AmpC and carbapenem-hydrolysing β-lactamases. J Antimicrob Chemother.

[37] Livermore DM, Mushtaq S, Warner M, Vickers A, Woodford N (2017). In vitro activity of cefepime/zidebactam (WCK 5222) against Gram-negative bacteria. J Antimicrob Chemother.

