# Supplementary material for: Multidrug-resistant Gram-negative clinical isolates with reduced susceptibility/resistance to cefiderocol: which are the best present and future therapeutic alternatives?
Source: Eur J Clin Microbiol Infect Dis. 2023 Dec 14;43(2):339–54. doi: 10.1007/s10096-023-04732-4 (PMC10821827; doi:10.1007/s10096-023-04732-4)
Supplement: Supplementary file 1 — (DOCX 35 kb) [file 10096_2023_4732_MOESM1_ESM.docx]

| Type of Carbapenamase produced |  | Cumulative % of isolate at MIC (mg/L) | | | | | | | | | | | | % of susceptible isolates^b^ | MIC_50_ | | MIC_90_ | | Range | |
| --- | --- | --- | --- | --- | --- | --- | --- | --- | --- | --- | --- | --- | --- | --- | --- | --- | --- | --- | --- | --- |
|  | BL/BLI combinations^a^ | ≤0.125 | 0.25 | 0.5 | 1 | 2 | 4 | 8 | 16 | 32 | 64 | ≥128 |  | |  | | |  | |  |
| NDM-type  n = 56 | FDC |  |  |  | 21.4 | 46.4 | 55.4 | 64.3 | 73.2 | 85.7 | 91.1 | 100 | 46.4 | | | 4 | | 64 | | ≤0.25 >256 |
|  | ATM |  | 3.6 | 7.1 | 7.1 | 8.9 | 8.9 | 8.9 | 12.5 | 16.1 | 32.1 | 100 | 7.1 | | | 256 | | 256 | | ≤0.25 >256 |
|  | AZA | 33.9 | 42.9 | 48.2 | 62.5 | 75 | 78.6 | 83.9 | 87.5 | 91.1 | 91.1 | 100 | 62.5 | | | 1 | | 32 | | ≤0.125 >128 |
|  | FEP |  |  |  |  |  |  | 1.8 | 3.6 | 7.1 | 10.7 | 100 | 0 | | | 256 | | 256 | | ≤0.25 >256 |
|  | FEP-T | 1.8 | 3.6 | 12.5 | 17.9 | 25 | 32.1 | 39.3 | 48.2 | 60.7 | 83.9 | 100 | 17.9 | | | 32 | | 128 | | ≤0.125 >128 |
|  | FEP-Z | 82.1 | 87.5 | 87.5 | 92.9 | 92.9 | 92.9 | 92.9 | 92.9 | 92.9 | 92.9 | 100 | 92.9 | | | 0.125 | | 1 | | ≤0.125 >128 |
|  | IMP |  |  |  | 3.6 | 5.4 | 21.4 | 51.8 | 73.2 | 80.4 | 91.1 | 100 | 5.4 | | | 8 | | 64 | | ≤0.25 >256 |
|  | I/R |  |  |  |  | 3.6 | 21.4 | 51.8 | 71.4 | 82.1 | 92.9 | 100 | 3.6 | | | 8 | | 64 | | ≤0.125 >128 |
|  | MEM |  |  |  | 1.8 | 7.1 | 16.1 | 25 | 41.1 | 53.6 | 76.8 | 100 | 7.1 | | | 32 | | 128 | | ≤0.25 >256 |
|  | MVB |  |  |  | 5.4 | 14.3 | 23.2 | 33.9 | 50 | 73.2 | 100 | 100 | 33.9 | | | 32 | | 64 | | ≤0.125 >128 |
|  | MER-NAC | 57.1 | 58.9 | 60.7 | 64.3 | 69.6 | 80.4 | 80.4 | 80.4 | 82.1 | 89.3 | 100 | 69.6 | | | 0.125 | | 128 | | ≤0.125 >128 |
|  | FDC-AVI | 28.6 | 39.3 | 44.6 | 53.6 | 58.9 | 67.9 | 71.4 | 83.9 | 87.5 | 91.1 | 100 | 58.9 | | | 1 | | 64 | | ≤0.25 >256 |
|  | FDC-REL | 19.6 | 33.9 | 42.9 | 53.6 | 58.9 | 67.9 | 80.4 | 89.3 | 91.1 | 96.4 | 100 | 58.9 | | | 2 | | 64 | | ≤0.125 >128 |
|  | FDC-VAB |  |  |  | 21.4 | 48.2 | 57.1 | 73.2 | 82.1 | 91.1 | 91.1 | 100 | 48.2 | | | 4 | | 32 | | ≤0.25 >256 |
|  | FDC-TAN | 28.6 | 50 | 55.4 | 67.9 | 71.4 | 80.4 | 85.7 | 91.1 | 94.6 | 94.6 | 100 | 71.4 | | | 0.5 | | 16 | | ≤0.125 >128 |
|  | FDC-ZID | 80.8 | 83.9 | 87.5 | 89.3 | 89.3 | 91.1 | 91.1 | 91.1 | 94.6 | 94.6 | 100 | 89.3 | | | 0.125 | | 4 | | ≤0.125 >128 |
|  | FDC-NAC | 46.4 | 58.9 | 66.1 | 73.2 | 78.6 | 83.9 | 85.7 | 91.1 | 92.9 | 94.6 | 100 | 78.6 | | | 0.25 | | 16 | | ≤0.25 >256 |
| VIM-type  n= 5 | FDC |  |  |  |  |  | 40 | 60 | 80 | 100 | 100 | 100 | 0 | | | 8 | | 32 | | ≤0.25 >256 |
|  | ATM |  |  |  |  |  |  |  |  |  |  | 100 | 0 | | | 256 | | 256 | | ≤0.25 >256 |
|  | AZA | 60 | 60 | 80 | 80 | 80 | 80 | 80 | 100 | 100 | 100 | 100 | 80 | | | 0.125 | | 16 | | ≤0.125 >128 |
|  | FEP |  |  |  |  |  |  |  |  |  | 20 | 100 | 0 | | | 256 | | 256 | | ≤0.25 >256 |
|  | FEP-T | 40 | 60 | 60 | 100 | 100 | 100 | 100 | 100 | 100 | 100 | 100 | 100 | | | 16 | | 128 | | ≤0.125 >128 |
|  | FEP-Z | 80 | 80 | 80 | 100 | 100 | 100 | 100 | 100 | 100 | 100 | 100 | 100 | | | 0.125 | | 128 | | ≤0.125 >128 |
|  | IMP |  |  |  |  |  |  | 20 | 60 | 100 | 100 | 100 | 0 | | | 16 | | 32 | | ≤0.25 >256 |
|  | I/R |  |  |  |  |  | 20 | 20 | 60 | 80 | 100 | 100 | 0 | | | 16 | | 64 | | ≤0.125 >128 |
|  | MEM |  |  |  |  |  | 20 | 40 | 40 | 80 | 80 | 100 | 0 | | | 32 | | 128 | | ≤0.25 >256 |
|  | MVB |  |  |  |  |  | 40 | 40 | 60 | 80 | 100 | 100 | 40 | | | 16 | | 64 | | ≤0.125 >128 |
|  | MER-NAC | 60 | 60 | 60 | 60 | 80 | 80 | 80 | 80 | 80 | 100 | 100 | 80 | | | 0.125 | | 128 | | ≤0.125 >128 |
|  | FDC-AVI | 40 | 60 | 60 | 80 | 80 | 80 | 100 | 100 | 100 | 100 | 100 | 80 | | | 0.25 | | 8 | | ≤0.25 >256 |
|  | FDC-REL | 20 | 40 | 40 | 60 | 60 | 60 | 80 | 100 | 100 | 100 | 100 | 60 | | | 1 | | 16 | | ≤0.125 >128 |
|  | FDC-VAB |  |  |  |  |  | 40 | 60 | 80 | 100 | 100 | 100 | 0 | | | 8 | | 32 | | ≤0.25 >256 |
|  | FDC-TAN | 40 | 60 | 60 | 100 | 100 | 100 | 100 | 100 | 100 | 100 | 100 | 100 | | | 0.25 | | 1 | | ≤0.125 >128 |
|  | FDC-ZID | 80 | 80 | 80 | 100 | 100 | 100 | 100 | 100 | 100 | 100 | 100 | 100 | | | 0.125 | | 1 | | ≤0.125 >128 |
|  | FDC-NAC | 40 | 60 | 60 | 60 | 60 | 60 | 80 | 100 | 100 | 100 | 100 | 60 | | | 0.125 | | 16 | | ≤0.25 >256 |

**Table S1.** Cumulative MIC distribution of novel BL/BLI commercially-available or under clinical development based on aztreonam, cefepime, imipenem and meropenem, and cefiderocol in the presence or absence of avibactam, relebactam, vaborbactam, zidebactam, taniborbactam, and nacubactam for 61 cefiderocol-reduced susceptibility/resistant- *Enterobacterales* strains producing NDM- or VIM-like enzymes

^a^Antibiotic abbreviations; ATM, aztreonam; FDC, cefiderocol ; AZA, aztreonam/avibactam; FEP, cefepime; FEP-T, cefepime/taniborbactam ; FEP-Z, cefepime/zidebactam; IMP, imipenem; I/R, imipenem/relebactam; MEM, meropenem; MVB, meropenem/vaborbactam ; MEM-NAC, meropenem/nacubactam; FDC-AVI, cefiderocol/avibactam; FDC-REL, cefiderocol/relebactam ; FDC-VAB, cefiderocol/vaborbactam ; FDC-TAN, cefiderocol/taniborbactam ; FDC-ZID, cefiderocol/zidebactam ; FDC-NAC, cefiderocol/nacubactam; the concentration of ß-lactamase inhibitors were fixed at 4mg/L for avibactam, relebactam, taniborbactam, zidebactam, nacubactam and durlobactam. The concentration of vaborbactam was fixed at 8mg/L. ^b^ According to EUCAST if the breakpoint is available, otherwise according to the respective ß-lactam in the ß-lactam/ß-lactamase inhibitor combination, and according to cefiderocol EUCAST breakpoint defining susceptible strains as MIC value ≤ 2 mg/L for Enterobacterales
